# Time-explicit life cycle assessment: a flexible framework for coherent consideration of temporal dynamics

**DOI:** 10.1007/s11367-025-02539-3

**Published:** 2025-10-28

**Authors:** Amelie Müller, Timo Diepers, Arthur Jakobs, Giuseppe Cardellini, Niklas von der Assen, Jeroen Guinée, Bernhard Steubing

**Affiliations:** 1 Institute of Environmental Sciences (CML), Leiden University, P.O. Box 9518, Leiden, RA 2300 The Netherlands; 2 Flemish Institute for Technology Research (VITO), EnergyVille, Thor Park 8310, Genk, 3600 Belgium; 3 Institute of Technical Thermodynamics (LTT), RWTH Aachen University, Schinkelstrasse 8, Aachen, 52062 Germany; 4 Technology Assessment Group, Laboratory for Energy Analysis (LEA), Center for Nuclear Engineering and Sciences & Center for Energy and Environmental Sciences, Paul Scherrer Institute PSI, Forschungsstrasse 111, Villigen, 5232 Switzerland

**Keywords:** Temporal distribution, Temporal evolution, Dynamic LCA, Prospective LCA, Open-source software, Time-differentiated, Time-resolved, Dynamic modelling

## Abstract

**Purpose:**

A well-known limitation of conventional Life Cycle Assessment (LCA) is the lack of temporal considerations, particularly the temporal distribution and evolution of processes, emissions, and environmental responses. While these aspects have been explored to some extent in dynamic and prospective LCA, a comprehensive approach for considering both temporal distribution and evolution is currently missing. We introduce a novel framework for time-explicit LCA that integrates the temporal distribution and evolution of product systems in the Life Cycle Inventory (LCI) phase and supports dynamic characterization of emissions in the Life Cycle Impact Assessment (LCIA) phase.

**Methods:**

The proposed approach expands the conventional LCA matrices to incorporate timing and time-based changes. We use a best-first graph traversal to derive an absolute timeline of intermediate flows by convolving relative temporal distributions at the process level. These timings are then integrated into the LCA matrices by adding time-specific row-column pairs in the technology matrix. Temporal markets are used to distribute product demands to the most-suitable processes in time-specific background databases. New rows in the biosphere matrix represent time-specific elementary flows. By preserving the timing of elementary flows during inventory calculation, time-explicit LCA enables dynamic alongside conventional LCIA. The proposed framework can be used for assessing any product system and impact category. An implementation of time-explicit LCA is provided in the open-source python package *bw_timex*, part of the *Brightway* ecosystem.

**Results:**

We demonstrate the framework with a simplified case study of an electric vehicle (EV). For a Paris-Agreement-compatible scenario, which assumes strong decarbonization over time, time-explicit LCA determines the EV's total Global Warming Impact to be half that of a 2020 conventional LCA and nearly double that of a 2040 prospective LCA. These differences arise because time-explicit LCA uses time-specific inventory data for each timestep, depending on the timing of processes in the supply chain, contrasting the conventional or prospective cases, which rely on a single inventory database. To further demonstrate dynamic characterization, we show the instantaneous and cumulative radiative forcing over the EV life cycle.

**Conclusions:**

Overall, time-explicit LCA can provide more representative results compared to conventional LCA, by considering when processes and emissions occur and what the state of the systems is at these timings. This is particularly valuable for long-lived products in temporally variable or fast-evolving systems. Future research should focus on filling data gaps and connecting time-explicit LCA with spatial LCA or dynamic material flow analysis.

**Graphical Abstract:**

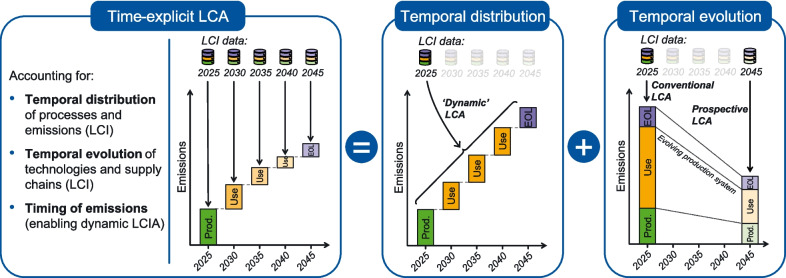

## Introduction

Like all models, life cycle assessment (LCA) models simplify the real world, reducing complexity to accommodate data and modelling constraints. One increasingly questioned simplification is that LCA typically treats processes, emissions, and environmental responses as static, disregarding temporal considerations (ISO 14040 2006). The importance of temporal considerations in LCA has been demonstrated in many studies and summarized in multiple reviews (Beloin-Saint-Pierre et al. 2020; Lueddeckens et al. 2020; Sohn et al. 2020; Su et al. 2021b). To structure the multitude of temporal considerations in both the life cycle inventory (LCI) and the life cycle impact assessment (LCIA) phases in LCA, we generally distinguish two categories: *temporal distribution* and *temporal evolution*.

### Temporal distribution

In the real world, supply chains must obey a certain temporal sequence, as products must be produced before they can be consumed. In other words, there is a time lag between demand and supply (Beloin-Saint-Pierre et al. 2014; Tiruta-Barna et al. 2016). This time lag can originate not only from the processes themselves taking a certain time to complete (e.g., a distinct process profile, such as a long use phase) but also from a delay between production and consumption (e.g., transport or storage processes) (Beloin-Saint-Pierre et al. 2014; Tiruta-Barna et al. 2016). Consequently, emissions and the induced environmental impacts are spread across time. We summarize temporal considerations that describe the timing of processes, emissions, and environmental responses under the term *temporal distribution*.

Conventional LCA typically does not model the temporal distribution of real-world systems, arguing that this simplification does not significantly influence a study’s outcomes. Instead, it implicitly assumes that the entire system occurs in the present moment, which Arvidsson et al. (2023) describe as the “ever-advancing ‘now’.” This reference to current time in conventional LCA is often implied by the presumed representativeness of contemporary conditions in the data (Guinée et al. 2002). A frequently used term for LCAs that account for aspects of temporal distribution is dynamic LCA (dLCA), although this term has been used inconsistently (Beloin-Saint-Pierre et al. 2020; Lueddeckens et al. 2020; Sohn et al. 2020; Su et al. 2021a). As a key characteristic, dLCA retains the timing of LCIs, e.g., emission $x$ occurring at time $t$, with various methods proposed to calculate the temporal sequence of inventories. Beloin-Saint-Pierre et al. (2014) propose the ESPA (enhanced structural path analysis) approach, which models the temporal distribution of intermediate and elementary flows using “process-relative temporal distributions” (rTDs). Dynamic inventories are derived through convolution and power-series-expansion. Building on this, Cardellini et al. (2018) also apply convolution of rTDs but prioritize processes using a “best-first” graph traversal algorithm (i.e., traversing processes with the highest impacts first), implemented in the tool *Temporalis* (Cardellini and Mutel 2018). Tiruta-Barna et al. (2016) propose to use the technosphere matrix as an adjacency matrix and calculate the temporal sequence of processes using a supply-demand model. This allows, in contrast to rTDs, to directly model global process behavior, such as process durations and production profiles. This approach has been operationalized in the tool *DyPLCA* (Pigné et al. 2020). Next to the timing of LCIs, dLCA also investigates time-dependencies of the environmental responses at the LCIA phase, often referred to as dynamic LCIA. Various approaches to dynamic characterization have been developed for the impact categories climate change (Levasseur et al. 2010; Kendall 2012; Shimako et al. 2016; Tiruta-Barna 2021; Lan and Yao 2022; Ventura 2022), including different indicators, such as global warming potential (GWP) and global mean temperature change (GMTC). Dynamic characterization for other impact categories is less common but has been studied for air pollution (Shah and Ries 2009), toxicity (Lebailly et al. 2014; Shimako et al. 2017), noise (Cucurachi and Heijungs 2014), and water use (Núñez et al. 2015). Existing dLCAs usually focus on climate change impacts, often covering biogenic carbon in bio-based materials (Levasseur et al. 2012b, 2012a; Brandão et al. 2013; Shimako et al. 2016), the built environment (Breton et al. 2018), transport (Albers et al. 2019), and CO_2_-based products (von der Assen et al. 2013). While the aforementioned studies consider the timing of emissions and apply dynamic characterization, they still model a steady-state operation of processes within a static supply chain configuration and a steady-state environment, assuming that omitting temporal evolution at LCI and LCIA is a reasonable modelling simplification.

### Temporal evolution

In reality, processes, supply chains and the state of the environment change over time. These changes may originate from variations in process operation (e.g., temporal profile of solar power production), structural shifts in supply chains (e.g., integration of novel renewable technologies into the electricity mix), or changing background conditions in the environment (e.g., increasing abundance of CO_2_ in the atmosphere). We summarize the time-based changes in processes, emissions and environmental responses under the term *temporal evolution.*

Capturing the temporal evolution towards *future* systems is central to the field of prospective LCA (pLCA). A pLCA “models the product system at a future point in time relative to the time at which the study is conducted” (Arvidsson et al. 2023). Various methods are used in pLCA studies to adapt LCIs based on projections for the future developments of product systems (Thonemann et al. 2020). While early pLCA studies mainly focused on the projection of the technology under review (foreground system), recent studies have shifted towards modeling economy-wide projections (foreground and background system) (Mendoza Beltran et al. 2020; Sacchi et al. 2022). Concerning existing software for prospective data generation, *premise* (Sacchi et al. 2022) has emerged as a widely used tool to modify ecoinvent databases based on integrated assessment model output. The focus of pLCA studies is typically on changes at the LCI stage, while temporal evolution at the LCIA stage is rarely considered. Regardless of projection methods and scope, pLCA approaches have in common that they model a system as a *snapshot* at distinct future points in time. These snapshots can be viewed as prospective *static* LCAs: the entire production system is moved forward in time, but all processes in the system are still simplified to happen simultaneously at this future timestep, under these future steady-state conditions. This means that any temporal distribution effect is left unaccounted. A conceptually similar approach to pLCA is retrospective or historical LCA, which uses past data rather than future projections to adapt LCIs (Arvidsson et al. 2023; Bruhn et al. 2024). However, like pLCA, retrospective LCA typically produces steady-state snapshots in time, without consideration of temporal distribution. 

Table 1 summarizes how existing LCA methods treat temporal distribution and evolution at the LCI and LCIA phase.

**Table 1:** Schematic overview of how different LCA methods typically treat temporal distribution and temporal evolution at the life cycle inventory (LCI) and life cycle impact assessment (LCIA) phase

	**Life cycle inventory**		**Life cycle impact assessment**	
	Temporal distribution	Temporal evolution	Temporal distribution	Temporal evolution
Conventional LCA	No, 1 current timestep	No	No	No
Dynamic LCA	Yes, multiple timesteps	Rarely	Yes	No
Prospective LCA	No, 1 future timestep	Yes	No	Rarely
Retrospective LCA	No, 1 past timestep	Yes	No	No
Time-explicit LCA (*proposed in this study*)	Yes, multiple timesteps	Yes	Yes	Yes

### Joint consideration of temporal distribution and evolution

In current literature, temporal distribution and evolution are mostly considered separately. While dLCA studies emphasize that systems are temporally distributed, they rarely account for their temporal evolution. Conversely, pLCA studies consider the temporal evolution of technologies and associated emissions at a future point in time but do not consider that processes and emissions are also distributed over time, see Table 1. Although a consistent treatment of temporal dynamics is widely recognized as important (Beloin-Saint-Pierre et al. 2020), it is often constrained by the limited availability of tools and data to address both aspects simultaneously (Vance et al. 2022).

Existing work that jointly considers temporal distribution and evolution usually focuses on a subset of inputs, e.g., electricity supply, and only considers the temporal distribution and evolution of this subset, but not other inputs or any upstream supply chains, or targets a single sector (e.g., buildings), while a generalizable and transparent method is missing. In a seminal early work, Collinge et al. (2013) conduct a dLCA of an institutional building and add yearly inventories for fuel and electricity and yearly emission factors to the foreground system. Zimmermann et al. (2015) add yearly prospective electricity mixes during the use phase in a pLCA study on electric mobility in Germany but apply static LCIA methods, using the term “time-resolved LCA.” A similar approach, but including static and dynamic LCIA for climate change, has been conducted by Peng et al. (2019) for a case study on compressors in China, using system dynamics to calculate yearly prospective electricity mixes. Reinert et al. (2021) optimize the costs of an energy system transition and then determine environmental impacts using different prospective databases based on the optimized deployment time of processes. Sigüenza et al. (2021) developed a time-vintage LCA model that splits the product system into life cycle stages and calculates different foreground and background LCIs per life cycle stage for each model cohort. Bruhn et al. (2023) argue that pLCAs for long-lived products, such as the built environment, should use data from different projection years for the different life cycle stages. Beloin-Saint-Pierre et al. (2016) use a systematic method (ESPA, cf. Beloin-Saint-Pierre et al. (2014)) to account for the temporal distribution of fore- and background processes, linking a subset of processes to their temporal evolution and applying dynamic LCIA. However, the linking to the temporal evolution of processes required extensive manual work and their excel-based workflow is not publicly available. Negishi et al. (2018) and Negishi et al. (2019) present a notable example of joint consideration of temporal distribution and evolution in LCI and LCIA for the building sector. They link a static building model to a dynamic parameter database that models time-based changes at the building (e.g., performance degradation), user (e.g., occupant behavior), and system (e.g., energy mix) levels. Foreground processes are discretized into fixed time intervals (e.g., 1 or 10 years), during which parameters are assumed constant. This inventory is then processed in the *DyPLCA* tool (Pigné et al. 2020), connecting the foreground processes to the data of the dynamic parameter database and adding the temporal distribution of supply chain processes. Finally, the resulting dLCI is characterized with dynamic LCIA for three climate change indicators. While these two studies are a substantial step towards temporal coherence in LCI and LCIA, the underlying algorithm in *DyPLCA* is not made publicly accessible, hindering the comparison to our approach. Lastly, recent work on coupling dLCA and pLCA for assessing transition paths in a tool called *Prosperdyn* seems promising (Lang-Quantzendorff and Beernmann 2024), but at the time of writing no published information could be found on the tool. Although these approaches highlight the importance of jointly accounting for temporal distribution and evolution of processes, emissions, and environmental responses in LCAs, they have limitations, such as a lack of transparency, focus on only specific sectors, a subset of processes or life cycle assessment steps, fixed temporal scopes and scalability constraints. As outlined above, existing tools such as *Temporalis* and *DyPLCA* support modeling the temporal distribution of processes and emissions, while tools like *premise* enable the projection of technological evolution at discrete points in time. Although some case-specific implementations, such as Negishi et al. (2019), combine both aspects to a degree, no existing tool offers a generalizable, transparent, and scalable solution that accounts for both temporal distribution and evolution simultaneously, which is essential for time-explicit LCA.

We propose a novel framework to simultaneously account for temporal distribution and temporal evolution in LCA by both considering the timing of processes and emissions as well as the state of technologies and supply chains at the respective point in time. We coin this framework “time-explicit LCA.” An implementation is available in the open-source python package *bw_timex* (Diepers et al. 2025b), which is part of the *Brightway* LCA ecosystem (Mutel 2017). In the following section, the framework is described and demonstrated with a case study of an electric vehicle (EV). We show that time-explicit LCA can yield more representative results for environmental impacts, particularly for temporally variable, fast-evolving systems or long-lived products with impacts spread considerably over their lifetime.

## Method

We first introduce the mathematical basis of time-explicit LCA. Then, we describe the time-explicit LCA framework and demonstrate it with a simple system. An in-depth description of the software implementation is given in Diepers et al. (2025b).

### Mathematical basis

The conventional inventory problem in LCA is described by Eq. (1)(Heijungs and Suh 2002):

1 $$h=C B { A}^{-1} f$$

where:

- ${f}_{(\mathrm{products} \times 1)}$ is the demand vector of the functional unit,
- ${A}_{(\text{products }\times \text{ processes})}$ is the technology matrix, whose element ${a}_{k,p}$ represents the amount of product $k$ required or produced by process $p$,
- ${B}_{\left(\text{elementary flows }\times \text{ processes}\right)}$ is the intervention or biosphere matrix, whose element ${b}_{j,p}$ represents elementary flow $j$ (e.g., emission or resource use) emitted or consumed by process $p$,
- ${C}_{\left(\text{impact categories }\times \text{ elementary flows}\right)}$ is the characterization matrix, whose element ${c}_{i,j}$ represents the characterization factor of elementary flow $j$ for impact category $i$, and
- ${h}_{\left(\text{impact categories }\times 1\right)}$ is the vector of environmental impacts.

Conventional LCA simplifies the complexity of real-world systems to a steady state in production technologies ($A$), their elementary flows ($B$) and translation to impacts ($C$) (Heijungs and Suh 2002). Existing temporal variation in data is handled by integrating it over time, leaving only an implicit reference to time through the temporal representativeness of the data (Guinée et al. 2002).

pLCA explicitly references time by modeling the system at a distinct future point $t$, described by Eq. (2).

2 $${h}_{t}={C}_{t}{ B}_{t}{ A}_{t}^{-1}{ f}_{t}$$

where: *t* represents a future point in time, e.g., year 2045.

pLCAs typically modify the $A$ and $B$ matrices to reflect the projected state of the technology at the future point in time (Mendoza Beltran et al. 2020; Thonemann et al. 2020; Sacchi et al. 2022). The issue is that pLCA treats the entire system as occurring at a single future point, ignoring that also a future system has temporally distributed processes and emissions. This corresponds to essentially performing a conventional, static LCA with projected data for one point in time. Retrospective LCA is conceptually the same, only for distinct points of time in the past.

Collinge et al. (2013) propose a mathematical formulation to account for temporal evolution across distinct timesteps, see Eq. (3):

3 $$h=\sum\nolimits_{{t}_{0}}^{{t}_{e}}{C}_{t} {B}_{t} {A}_{t}^{-1} {f}_{t}$$

where:

- $t$ represents a distinct point in time at which the state of the system is known, and
- ${t}_{0}$ and ${t}_{e}$ represent the start and end time points of the analysis, usually the beginning and ending of the product or system life cycle (Collinge et al. 2013).

Equation 3 is in essence the sum of Eq. 2 for all points in time with available data. This means that the LCA equation is evaluated separately for each timestep: The system is split into temporal segments, each with its own distinct set of technology, biosphere and characterization matrices. While this improves upon modeling a system only at a single current (Eq. 1) or future (Eq. 2) point in time, it still neglects interconnections across timesteps.

For example, consider an EV life cycle: Collinge et al. (2013) split the life cycle into distinct timesteps, e.g., car factory construction at ${t}_{0}$, car assembly at ${t}_{1}$, car use phase from ${t}_{2}$ to ${t}_{n-1}$, and disposal at ${t}_{n}$. According to Eq. 3, each segments’ supply chain is modeled at the same time as the segment itself. For example, materials for factory construction are produced at ${t}_{0}$ and all car components at ${t}_{1}$. However, in reality, these activities occur sequentially–materials required for the factory need to be produced before the factory can be built, and so on. Such time lags exist throughout supply chains, leading to complex temporal distributions in real-world systems. Collinge et al. (2013) acknowledge this limitation, noting that “a more complete formulation would involve specifying the lag time for each supply–demand linkage, which would require calculation using a tree structure rather than a matrix structure, as the number of inputs at different time lags would multiply with each step back through the supply chain” (Collinge et al. 2013, p.4).

In time-explicit LCA, the results of a tree-based time lag propagation are used to extend the original matrices. This expansion allows us to reflect the timing of processes and emissions in the supply chain (temporal distribution) and, at the same time, to consider different process inventories for different points in time (temporal evolution). The resulting mathematical formulation of time-explicit LCA is structurally identical to Eq. 1 but with temporally extended matrices, as denoted by the asterisks (*), see Eq. (4):

4 $${h}^{*}={C}^{*}{B}^{*}{{A}^{*}}^{-1}{f}^{*}$$

where:

- ${f}_{\left(\text{products }@\text{ timesteps }\times 1\right)}^{*}$ is the time-explicit demand vector of the functional unit,
- ${A}_{(\text{products }@\text{ timesteps }\times \text{ processes }@\text{ timesteps})}^{*}$ is the time-explicit technology matrix, whose element ${a}_{k,p}^{*}$ represents the amount of product $k$ at a specific timestep required or produced by process $p$ at a specific timestep,
- ${B}_{(\text{elementary flows }@\text{ timesteps }\times \text{ processes }@\text{ timesteps})}^{*}$ is the time-explicit biosphere matrix, whose element ${b}_{j,p}^{*}$ represents elementary flow $j$ at a specific timestep emitted or consumed by process $p$ at a specific timestep,
- ${C}_{\left(\text{impact categories }@\text{ timesteps }\times \text{ elementary flows }@\text{ timesteps}\right)}^{*}$ is the time-explicit characterization matrix, whose element ${c}_{i,j}^{*}$ represents the characterization factor for impact category $i$ at a specific timestep for elementary flow $j$ at a specific timestep, and
- ${h}_{\left(\text{impact categories }@\text{ timesteps }\times 1\right)}^{*}$ is the vector of time-explicit environmental impacts.

The key distinction of the time-explicit LCA formulation is its ability to embed temporal information directly into the LCA matrices by adding a new element (row-column pair) for each process at a specific time (see section 2.2 for details). This expansion approach allows the elements of each matrix to correspond to different points in time. By contrast, conventional LCA (Eq. 1) assumes that matrix elements represent an implicitly defined “current” time, while pLCA (Eq. 2) and dLCA (Eq. 3) assume a single, fixed point in time–at a single future time or for each $t$ within the summation, respectively.

### Time-explicit LCA framework

**Fig. 1:**
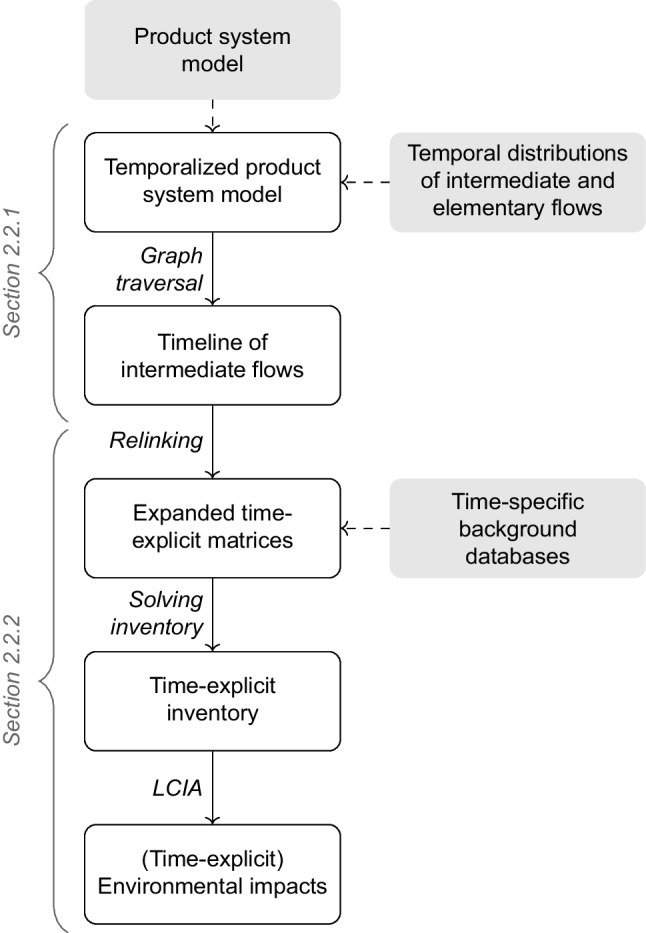
Overview of the time-explicit LCA framework

The implementation of the time-explicit LCA framework (Eq. 4) is outlined in the following section. Figure 1 provides an overview of the steps involved in a time-explicit LCA.

**Fig. 2:**
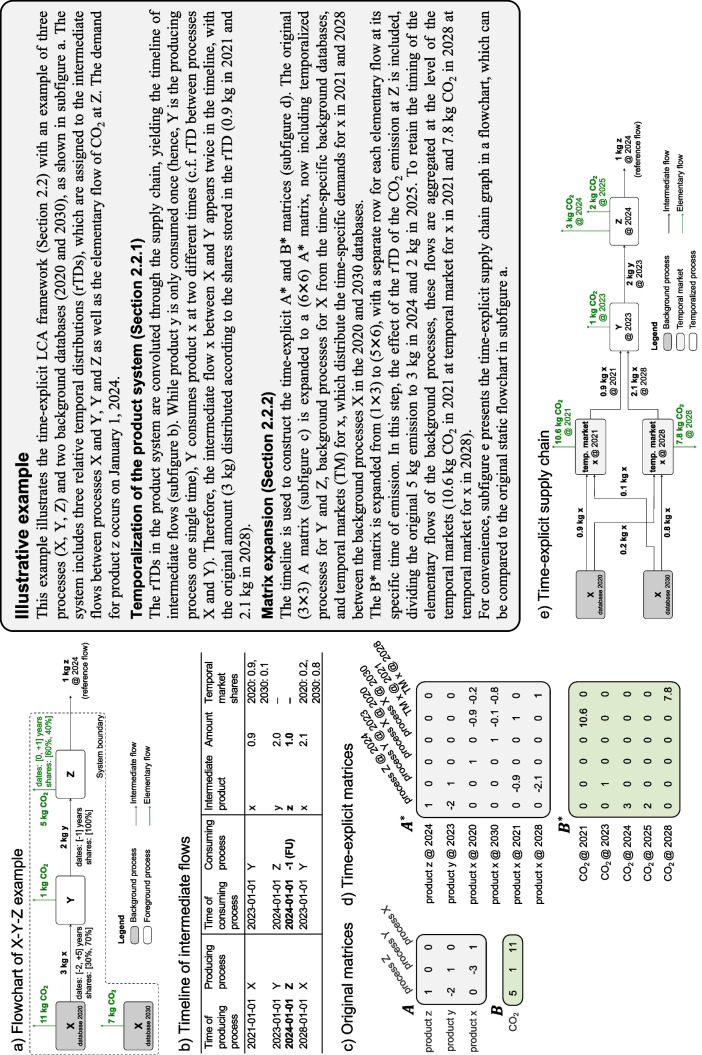
Time-explicit LCA procedure for an illustrative example consisting of three processes X, Y, and Z

First, we describe how a product system is temporalized (see section 2.2.1). Then, we explain how this temporal information is incorporated into the matrix structure (see section 2.2.2). The approach is demonstrated for a simple example in Fig. 2.

#### Temporalization of product systems

A time-explicit LCA must be informed of the absolute timing of processes across the system (temporal distribution) before linking them to their temporal evolution at these points in time. By absolute timing, we refer to the specific point in calendar time (i.e., the date) at which a process occurs, rather than just its relative temporal position within the product system. This absolute timing can be determined by propagating temporal information at the process level along the supply chain (Beloin-Saint-Pierre et al. 2014). For this purpose, we use rTDs as implemented in Cardellini et al. (2018). A rTD reflects how the total amount of a flow is distributed across time. For intermediate flows, rTDs describe when product $k$ is demanded relative to the timing of its consuming process $p$, and for elementary flows when elementary flow $b$ is emitted or consumed relative to the timing of its emitting or consuming process $p$. As time in LCA is inherently discrete (Heijungs and Suh 2002), continuous inputs or emissions can be discretized by sampling the supply or emission functions at specific intervals.

To determine the absolute timing of all processes and emissions, the rTDs are convolved along the supply chain. This procedure begins at the functional unit, which is demanded at an absolute point in time defined by the LCA practitioner. From there, the supply chain graph is traversed and the rTDs are propagated through time using convolution, following the approach of Cardellini et al. (2018). The best-first traversal algorithm of Cardellini et al. (2018) is more suitable than a breadth-first (Beloin-Saint-Pierre et al. 2014) or depth-first variant (Beloin-Saint-Pierre et al. 2014; Tiruta-Barna et al. 2016) as it prioritizes the traversal of the most important contributors, covering the relevant parts of the supply chain faster. For a detailed explanation of this traversal algorithm, readers are referred to the original work. Unlike Cardellini et al. (2018), we construct an absolute timeline of intermediate flows, rather than that of elementary flows. This timeline of intermediate flows specifies the absolute timing of the producing and consuming process for each intermediate flow in the product system. The rTDs of an exemplary system and the resulting timeline of intermediate flows are shown in Fig. 2a and b.

In addition to the absolute timing of processes, a time-explicit LCA requires information on the temporal evolution of processes over time. This is achieved using time-specific background databases. Time-specific is defined here as referring to a single absolute point in time. Time-specific databases, thus, represent the state of the production system (temporal evolution) at single absolute points in time, which is stored as metadata. The linking of intermediate flows to these time-specific inventory databases is described in the next section.

#### Matrix expansion

To reference to multiple time points in a single matrix, we build on the approach by Lesage et al. (2019). Each process at a specific time, derived from the timeline of intermediate flows, is treated as a separate process, referred to as a “temporalized process.” These temporalized processes are added as new columns to the ${A}^{*}$-matrix. Correspondingly, new rows are added for the “temporalized products” produced by these processes. To control the desired level of detail of new entries, the temporal resolution of the new entries can be harmonized by grouping them, e.g., on a yearly resolution.

**Fig. 3:**
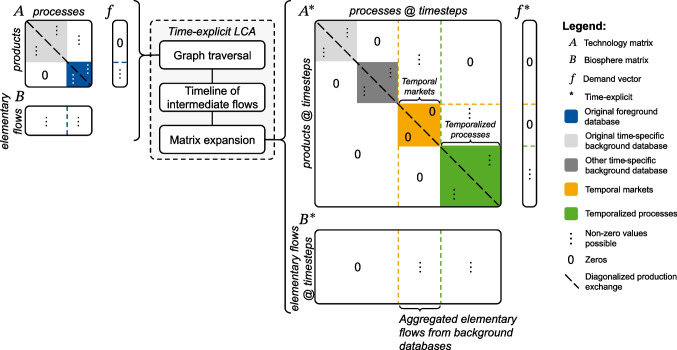
Construction of the time-explicit matrices ${\mathrm{A}}^{*}$ and ${\mathrm{B}}^{*}$ from the conventional (static) matrices $\mathrm{A}$ and $\mathrm{B}$

If a temporalized process receives an intermediate flow from a background database, the temporalized process is linked to the producing process(es) of the intermediate flow from the most temporally appropriate background database(s). This is achieved by introducing a new set of row-column pairs, called “temporal markets.” In traditional LCA, market processes distribute a demand for a product across spatial or technological alternatives (Wernet et al. 2016). In analogy, temporal markets distribute a demand across time, linking to processes that represent different temporal evolutions. They allocate this demand across time using temporal weighting factors based on the temporal proximity between the time of the producing process and the times of the most temporally appropriate background databases (see “temporal market shares” in timeline of intermediate flows in Fig. 2b). The default option is linear interpolation, which is consistent with common LCA practice and methods used in prospective background database generation. However, the option to take only the value from the nearest time-specific background database is also available, and users can implement custom interpolation methods if non-linear dynamics are more appropriate for their case or reflect non-linearities through more finely resolved time-specific background databases. A schematic representation of a time-explicit ${A}^{*}$-matrix compared to a conventional $A$-matrix is given in Fig. 3. The time-explicit matrices for the simple example are shown in Fig. 2d.

Next, the biosphere matrix ${B}^{*}$ is also reconstructed to retain the temporal information at the level of elementary flows. The timing of an elementary flow is determined by the timing of its emitting process, convolved with the rTD of the elementary flow, if available. This accounts for any additional temporal shift of the elementary flow relative to the emitting process, e.g., long-term emissions from landfills. Thus, the timesteps in the time-explicit ${B}^{*}$-matrix can differ from those in the time-explicit ${A}^{*}$-matrix_,_ as demonstrated for the example in Fig. 2d. To retain the correct timing of the elementary flows, elementary flows from processes in the background databases are aggregated at the corresponding temporal market, resulting in zero entries in ${B}^{*}$ for background processes, see Fig. 3. The resulting ${B}^{*}$-matrix is typically highly sparse due to the large number of timesteps.

Lastly, the time-explicit LCIs are obtained by multiplying the time-explicit biosphere matrix ${B}^{*}$ by the time-explicit supply vector ${s}^{*}={{A}^{*}}^{-1}\cdot {f}^{*}$, following conventional matrix-based LCA calculation. The time-explicit LCI retains temporal information of the emissions, enabling subsequent characterization with either conventional characterization factors or dynamic characterization functions. Dynamic characterization functions for the climate change metrics radiative forcing and dynamic GWP (Levasseur et al. 2010) are available in the *Brightway* library *dynamic_characterization* (Brightway 2025c). A simple software interface enables users to easily change the time horizon of the assessment and whether the time horizon should be treated as fixed or moving (Ventura 2022). In contrast to current examples of dynamic LCIA, full time-explicit LCIA would require to also consider the temporal evolution of environmental responses, e.g., due to changes of future greenhouse gas (GHG) background concentrations for GWP.

### Software implementation

The time-explicit LCA framework is implemented in the open-source Python software package *bw_timex* (Diepers et al. 2025b). *bw_timex* is part of the open-source LCA ecosystem *Brightway* (Mutel 2017), a widely used LCA software in the scientific community due to its flexibility and computational efficiency. The best-first graph traversal algorithm used in *bw_timex* originates from Cardellini et al. (2018), but has been updated and moved to the *Brightway* library *bw_graph_tools* (Brightway 2025a). The dynamic impact assessment methods are sourced from the *Brightway* library *dynamic_characterization* (Brightway 2025c). Additional information and instructions are available in the comprehensive and beginners-friendly online documentation of *bw_timex* (Diepers et al. 2025a)*.*

By making all source code publicly accessible and by relying exclusively on other open-source frameworks, this work contributes to a higher level of transparency, quality and productivity in the Industrial Ecology research community (Pauliuk et al. 2015).

## Case study

To demonstrate the capabilities of the developed framework, we apply time-explicit LCA in a case study of an EV. The product system is described in section 3.1. The case study results are presented in section 3.2. The full case study code is available as an annotated Jupyter Notebook in the *bw_timex* GitHub repository (Brightway 2025b).

### Case study setup

The goal of this case study is to assess a product system using time-explicit LCA and compare the results to those of a conventional LCA, a dLCA and a pLCA. Life cycles of EVs span several years and the climate change impact of EVs is highly sensitive to the electricity supply, which makes this a well-suited example for time-explicit LCA. We consider a cradle-to-grave model of an EV and assess the Global Warming Impact (GWI) over the EV’s lifetime using GWP100 from the Environmental Footprint 3.1 impact assessment method (European Commission 2023). To additionally showcase time-explicit LCA’s capability to reflect time-resolved environmental impacts, we calculate the resulting radiative forcing over time using dLCIA functions from the library *dynamic_characterization* (Brightway 2025c). The calculations for radiative forcing are based on Myhre et al. (2014), with updated numerical values for radiative efficiencies and substance lifetimes from the IPCC Assessment Report 6 (Smith et al. 2021). Further details are available in the Jupyter Notebook on GitHub (Brightway 2025b).

**Fig. 4:**
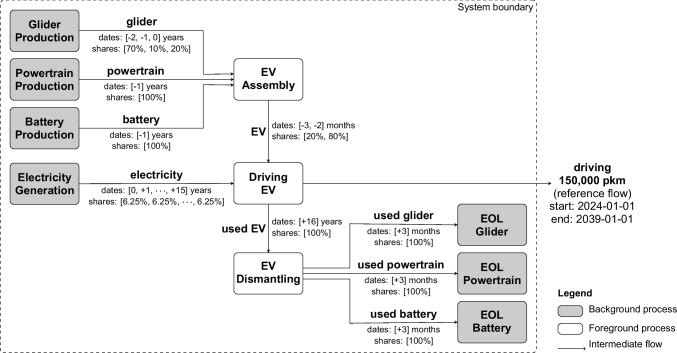
Flowchart of the EV modeled in the case study

To reduce complexity and focus on methodological implications rather than subject-specific findings, the modeled EV is greatly simplified. The product system is shown in Fig. 4. The foreground system consists of three processes covering the assembly, driving and dismantling of the EV. The foreground processes link to background processes from the ecoinvent 3.10 database (Wernet et al. 2016). For the background processes, we choose global average markets for the respective processes. The EV-specific parameter assumptions are listed in Table 2.

**Table 2:** Parameters choices for the EV

Parameter	Value
Vehicle lifetime	16 years
Mileage	150,000 km
Electricity consumption	0.2 kWh/km
Mass glider	840 kg
Mass powertrain	80 kg
Mass battery	280 kg

Figure 4 shows the product system as well as the rTDs embedded in the system. All intermediate flows that represent the production phase, i.e., flows between the EV assembly process and the background production processes, are dated back in time, e.g., 20% of the EV assembly occurs 3 months and 80% 2 months prior to the driving process. During the use phase, we assume a uniformly distributed electricity consumption across the EV’s lifetime. Finally, flows between the EV disassembly and the background end-of-life (EOL) treatment processes are dated forward in time, so that they occur after the EV’s lifetime. For the reference flow of the functional unit (FU), i.e., driving 150,000 person kilometers, we define the absolute starting time as January 1 st, 2024.

To represent temporal evolution, we provide three background databases representing the years 2020, 2030, and 2040. The prospective adjustments follow the IMAGE SSP2-RCP19 scenario (Stehfest et al. 2014). This scenario implies strong decarbonization efforts and was chosen to best showcase the differences introduced by the time-explicit LCA. The prospective databases are created with the ecoinvent 3.10 database using *premise* (Sacchi et al. 2022), implementing updates to only the electricity sector. For the reference cases of conventional and dynamic LCA, where the LCI data does not change over time, we use the background database representing the year 2020. As a result, the temporal information is fully omitted in the conventional LCA, and only the temporal distribution of the emissions is considered in the dynamic LCA, with unchanged LCI data.

### Results

The timeline of intermediate flows resulting from the convolution of rTDs with respect to the absolute starting time of the FU is shown in Table 3.

**Table 3:** Timeline of intermediate flows for the EV case study

Time of producing process	Producing process	Time of consuming process	Consuming process	Intermediate product	Amount^a^
2021–10-01	Glider Production	2023–10-01	EV Assembly	glider	117.6 kg
2021–11-01	Glider Production	2023–11-01	EV Assembly	glider	470.4 kg
2022–10-01	Glider Production	2023–10-01	EV Assembly	glider	16.8 kg
2022–10-01	Powertrain Production	2023–10-01	EV Assembly	powertrain	16 kg
2022–10-01	Battery Production	2023–10-01	EV Assembly	battery	56 kg
2022–11-01	Glider Production	2023–11-01	EV Assembly	glider	67.2 kg
2022–11-01	Powertrain Production	2023–11-01	EV Assembly	powertrain	64 kg
2022–11-01	Battery Production	2023–11-01	EV Assembly	battery	224 kg
2023–10-01	Glider Production	2023–10-01	EV Assembly	glider	33.6 kg
2023–10-01	EV Assembly	2024–01-01	Driving EV	EV	0.2 units
2023–11-01	Glider Production	2023–11-01	EV Assembly	glider	134.4 kg
2023–11-01	EV Assembly	2024–01-01	Driving EV	EV	0.8 units
**2024–01-01**	**Driving EV**	**2024–01-01**	**−1 (FU)**	**driving**	**150,000 pkm**
2024–01-01	Electricity Generation	2024–01-01	Driving EV	electricity	1875 kWh
2025–01-01	Electricity Generation	2024–01-01	Driving EV	electricity	1875 kWh
…	…	…	…	…	…
2039–01-01	Electricity Generation	2024–01-01	Driving EV	electricity	1875 kWh
2040–01-01	EV Dismantling	2040–01-01	Driving EV	used EV	1 unit
2040–04-01	EOL Battery	2040–01-01	EV Dismantling	used battery	280 kg
2040–04-01	EOL Powertrain	2040–01-01	EV Dismantling	used powertrain	80 kg
2040–04-01	EOL Glider	2040–01-01	EV Dismantling	used glider	840 kg

^a^Absolute supply amounts scaled to satisfy the FU

Bold indicates the FU process

**Fig. 5:**
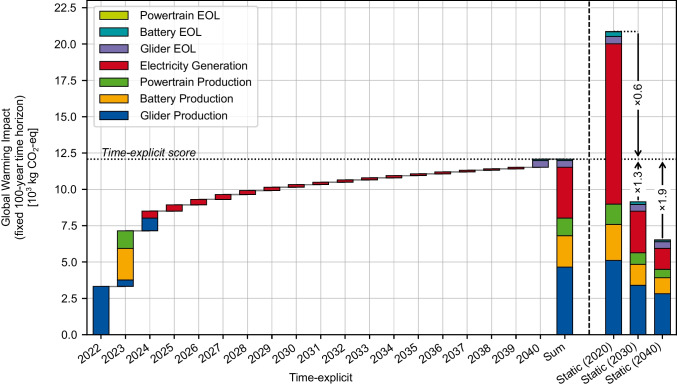
Time-explicit Global Warming Impact of the EV in comparison to static LCA results for different years

Figure 5 shows the GWI results of the EV life cycle of a time-explicit LCA compared to the results of static LCAs for the years 2020, 2030, and 2040. We apply GWP over a time horizon of 100 years, counting from the time of each emission. So, emissions in 2024 and 2040 are both characterized with GWP100. The total GWI in the time-explicit LCA amounts to 12 t CO_2_-eq, with the majority occurring at the beginning of the life cycle in 2022 to 2024 due to the production of the EV components. Over the EV’s use phase between 2024 and 2039, electricity consumption adds to the impacts. Even though the amount of electricity consumed by the EV is the same each year, the additional yearly impacts decrease with time because of the progressive decarbonization of the electricity sector in the considered scenario. After the EV’s lifetime, the EOL processes for the glider, battery, and powertrain cause a final increase in emissions.

Comparing the time-explicit GWI results with the results for the years 2020, 2030, and 2040 reveals that the total GWI for the time-explicit LCA is approximately half that of the 2020 static LCA but nearly twice that of the 2040 static LCA. For 2030, the static LCA yields 30% lower impacts than the time-explicit case. This is because the static LCA assumes that the whole system has reached the decarbonization level of 2030, while the time-explicit LCA assumes that each process has the decarbonization state of the specific timestep in which it occurs. For EV manufacturing–the largest impact contributor–the time-explicit LCA uses LCI data from 2022 to 2024, when relatively few decarbonization measures have been implemented, leading to higher impacts compared to projecting the manufacturing step to 2030. In contrast, EOL impacts in the time-explicit LCA are lower than those in the 2020 and 2030 case, as the more decarbonized 2040 state of technology is used for EOL processes in the time-explicit LCA. This effect is most pronounced for the battery EOL, where most of the impacts originate from electricity generation. Generally, we conclude that in the case of progressively decarbonizing background systems, static LCAs conducted at a prospective timestep in the middle of a product life cycle underestimate impacts early in the life cycle and overestimate impacts in the EOL. Time-explicit LCA, on the other hand, uses specific data for each timestep, reducing potential under- or overestimation.

**Fig. 6:**
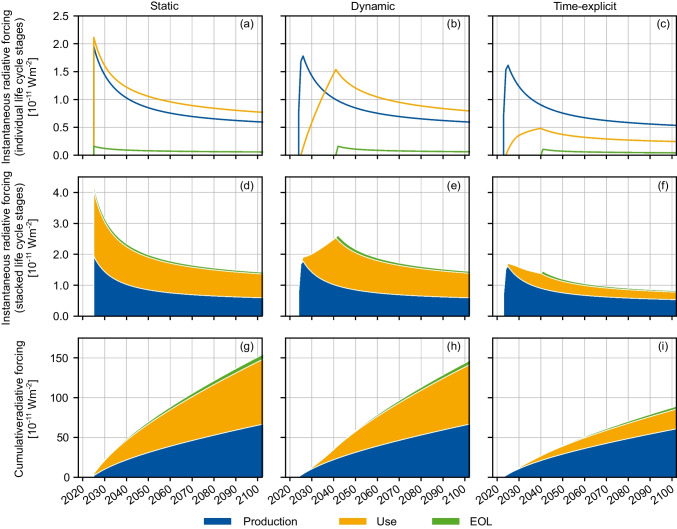
Instantaneous and cumulative radiative forcing over the EV life cycle, split by life cycle stage, comparing static (no temporal distribution, no temporal evolution), dynamic (only temporal distribution) and time-explicit (temporal distribution and evolution) LCA results. EOL = end-of-life

In addition to assessing the GWI using relative emission metrics such as GWP100, time-explicit LCA enables calculating the underlying radiative forcing caused by the emissions over the life cycle. Thereby, subjective choices of time horizons and reference gases can be avoided. Figure 6 shows the instantaneous (a–f) and cumulative (g–i) radiative forcing over the EV life cycle for static LCA (no temporal distribution, no temporal evolution), dynamic LCA (only temporal distribution), and time-explicit LCA (temporal distribution and evolution). The results are aggregated by the three life cycle stages: production, use phase, and EOL. For simplicity and ease of visual comparison, the starting year 2024 was chosen for all three cases. Integrating the instantaneous radiative forcing curves in Fig. 6 over a 100-year horizon starting from the time of emission and normalizing the result by the integrated radiative forcing of 1 kg CO_2_ over the same period reproduces the results in Fig. 5.

In the static case (Fig. 6a), all emissions occur at once in 2024, causing an immediate peak in radiative forcing that then gradually declines as the GHGs are processed by natural cycles. In the dynamic case (b), emissions occur at different times, lowering the overall peak because earlier emissions have partially decayed by the time later ones occur. This is visible in Fig. 6b, where particularly for the use phase, the radiation forcing curve flattens slightly over time, instead of increasing linearly. The time-explicit case (c) shows the combined effect of temporal distribution and temporal evolution and is, again, particularly evident for the use phase. As electricity supply decarbonizes, the radiative forcing curve flattens more drastically, reducing its peak to about one-third of that of the dynamic case, which does not consider decarbonization.

Stacking the instantaneous radiative forcing of the life cycle stages on top of each other (Fig. 6d-f) shows the dynamics over time for the whole product system. While the static case (d) reaches maximum radiative forcing at the beginning, since all emissions are assumed to occur at once, considering the timing of emissions in the dynamic case (e) shifts the maximum to the time of last EOL emission. Accounting for evolving LCIs (f) moves the maximum to the start of the use phase. After the start of the use phase, the natural decay of past emissions outweighs the additional forcing from emissions over time, which becomes progressively smaller due to the background decarbonization.

Lastly, Fig. 6g-i shows the cumulative radiative forcing. In the static case (g), all emissions occur at the beginning, leading to a steeper initial increase in cumulative radiative forcing compared to the dynamic case (h), in which some emissions are delayed, although the amount of each emission remains the same. The time-explicit case (i) shows the lowest cumulative radiative forcing, caused by the combined effect of delayed and reduced emissions. Overall, it is evident that the different considerations of the timing and emission amounts in static, dynamic and time-explicit LCA significantly change the resulting radiative forcing, both in terms of instantaneous and cumulative radiative forcing. This highlights the relevance of a dynamic LCIA, particularly when combined with time-explicit LCI. 

## Discussion

The proposed framework for time-explicit LCA offers several key advantages over existing methods. First and foremost, it presents a step towards a more representative modeling of real-world systems in LCA by simultaneously considering the temporal distribution and temporal evolution of processes and emissions over time. This closes a gap between the fields of dLCA and pLCA, which both cover temporal considerations to a certain extent.

To retrieve the timing of processes and emissions, rTDs of intermediate flows are convolved through the supply chain using a best-first graph traversal. Based on the resulting timeline of intermediate flows, the original LCA matrices are expanded, linking temporalized foreground processes to time-specific background processes in the time-explicit technology matrix, and maintaining the timing of elementary flows in the time-explicit biosphere matrix. By preserving the conventional mathematical formulation of the inventory problem (Heijungs and Suh 2002), we ensure compatibility with common tools for LCA calculations and existing methods for contribution and scenario analysis.

The proposed framework offers full flexibility of the underlying rTDs, which has been argued to be a key feature for the realization of time-resolved LCA (Beloin-Saint-Pierre et al. 2014). In addition, time-explicit LCA is versatile in the LCIA phase, enabling both static and dynamic impact assessment as the timing of emissions is reflected in the LCI. Generally, the choice of temporal resolution should align with the characteristics of the product system and the impact category considered. For example, yearly resolution is often sufficient when focusing on global process behavior or steady-state processes, and matches climate change indicators, where impacts are integrated over long time horizons. Finer resolutions (e.g., monthly or daily) may be needed when modeling systems with stronger temporal dynamics, such as seasonal production, and are particularly relevant for impact categories sensitive to short-term changes, such as water scarcity (Collet et al. 2014). The flexibility of the proposed framework supports this, accommodating various research goals depending on context and data availability.

To facilitate the application of the proposed framework by LCA practitioners, we provide an open-source implementation in the python package *bw_timex.* Users can provide (reusable) rTDs at the process level and time-specific background databases, and the algorithm automatically constructs the timeline, builds the time-explicit matrices and handles time-explicit LCI and LCIA calculations. Time-specific background databases can be seamlessly integrated from tools like *premise* (Sacchi et al. 2022) or provided by the users themselves. As part of the *Brightway* ecosystem (Mutel 2017), *bw_timex* can easily be combined with existing LCA workflows or tools from the *Brightway* family. Moreover, *bw_timex* could serve as a modular expansion to other matrix-based LCA software, given support for storing rTDs as additional data to intermediate and elementary flows and the support of an interface for data exchange (e.g., via the *randonneur* tool (Brightway 2025d)). As *bw_timex* retains the conventional matrix structure, the expanded time-explicit matrices could be exported back to the respective tool for further processing and analysis.

As we demonstrate in the EV case study, time-explicit LCA results differ from static results due to variations in the timing of processes, technological changes in background databases and the use of static or dynamic impact assessment methods. With more significant technological changes in the (projected) time-specific background systems (e.g., to meet ambitious climate targets), and supply chains that are spread over a long time (e.g., long-lived products with inputs in all life cycle stages), the time-explicit LCA results increasingly diverge from conventional LCA results. Contrarily, if the considered product system does not change over time, i.e., all time-specific background databases are the same, a time-explicit LCA results in an inventory identical to that of a dLCA, which is essentially the inventory of a static LCA distributed over time. Differences in the native resolution of the rTDs, as well as the optional temporal grouping may result in relinking to different time-specific inventories. This can potentially yield different time-explicit LCA results, similar to Beloin-Saint-Pierre et al. (2016), where switching between monthly and yearly resolution altered the preferred alternative.

### Old wine in new bottles?

The idea of incorporating temporal information in LCA by adding time-specific processes is not new. Heijungs and Suh (2002) first proposed this concept, comparing it to spatial LCA. However, to date it has seen limited systematic implementation, with only a few examples of manual addition of time-specific data (Collinge et al. 2013; Zimmermann et al. 2015; Beloin-Saint-Pierre et al. 2016; Peng et al. 2019). Heijungs and Suh (2002) have foreseen large data and computational requirements for such a temporalization as key bottlenecks. While these challenges remain only partially resolved (see next section on limitations), our implementation of *bw_timex* as an open-source library in the *Brightway* ecosystem (Mutel 2017) marks a significant step towards operationalizing this approach.

Many terms have been used in literature to describe temporal aspects in LCA, c.f. the terminology section in Beloin-Saint-Pierre et al. (2020). To clarify and avoid confusion with the widely used and sometimes misinterpreted term “dynamic LCA,” we propose the term “time-explicit LCA.” Time-explicit LCA aligns with what Beloin-Saint-Pierre et al. (2016) call “full dynamic LCA,” which they describe as the “system dynamics and impact assessment of a [temporally differentiated] LCI” (Beloin-Saint-Pierre et al. 2016) or—in our words—the consideration of both temporal distribution and evolution of processes and emissions at the LCI and LCIA stages.

Several existing tools already support individual aspects of time-based LCA, and not every use-case requires a fully time-explicit approach. These tools are well suited when the goal is to assess products and their supply chains at a single point in the future (*premise* (Sacchi et al. 2022)), to examine the influence of the timing of processing and emissions on the results (*Temporalis* (Cardellini et al. 2018)) or to explore supply–demand dynamics and system-wide delays (*DyPLCA* (Pigné et al. 2020)). The strength of *bw_timex* lies in providing an open-source computational framework that captures both the timing of processes and emissions and the evolution of technologies—without requiring manual mapping of processes to time-specific inventories. It supports various temporal resolutions, from hours to years, allowing researchers to tailor the time scale to the needs of their research question, impact category, and data availability. While *bw_timex* offers a unified, open-source framework for modelling systems across time, this level of detail may be unnecessary for simpler systems with few time-varying inputs. For example, a prospective LCA of the presented EV life cycle, that uses a time-averaged electricity mix for the use phase would offer a reasonable approximation, since electricity generation is the main source of climate change impacts in the system. Such a simplified temporal approach has been successfully applied to study time-based impacts of electric mobility in Šimaitis et al. (2025). However, for more complicated systems and for dynamic LCIA, the advantages of using *bw_timex* become more pronounced.

### Challenges for time-explicit LCA

Time-explicit LCA requires a substantial amount of data, much of which is currently not provided by LCI database providers. This includes data on (1) the temporal distribution of processes, (2) the temporal evolution of product systems and ideally (3) time-explicit LCIA. For point (1), while LCA practitioners usually know the rTDs of the foreground system, obtaining data on the temporal sequence of broader supply chains is more challenging. To address this, Pigné et al. (2020) have demonstrated that a supply-delay framework based on product groups can be used to temporalize entire databases, although at high computational costs. This temporal process categorization is implemented in the online tool *DyPLCA*, but unfortunately, the categorization itself is not publicly available, limiting its application to other tools. The time-explicit LCA framework proposed in this work could easily integrate alternative algorithms for determining process timing. Since the timeline of intermediate flows serves as the sole interface, the rest of the framework remains unaffected by the specific method used to generate temporal information. For point (2), advancements in prospective background data generation tools like *premise* (Sacchi et al. 2022) have made time-specific LCI data focusing on climate change mitigation scenarios in future years readily available. However, time-specific data for emissions relevant to other impact categories or at higher temporal resolution remain scarce. Historical LCA (Bruhn et al. 2024) is a step towards better data for past time-steps, while real-time data is gaining increasing traction in LCAs for the energy sector (Besseau et al. 2024). For point (3), time-explicit LCIA methods, covering both the timing and time-based changes in environmental responses, remain an emerging field and are limited to only a few impact categories. Existing work primarily focuses on adjusting the time horizon for impact calculations, such as modifying the GWP integral based on the time of emission (Levasseur et al. 2010; Ventura 2022). To additionally capture the temporal evolution in LCIA, the changing state of the environment needs to be accounted for, e.g., by considering increased future atmospheric background GHG concentrations.

Our implementation allows users to specify data for points 1 to 3 whenever available, while defaulting to static data if unavailable. For example, if no rTD is given for an intermediate flow between two processes, the producing process is assumed to happen at the same time as the consuming process. Or, if the background databases are only available at a different temporal resolution than the temporalized process, the temporal market for this process will interpolate between the nearest time-specific databases. Lastly, if no time-explicit impact assessment methods are provided, *bw_timex* will default to static LCIA methods. Thereby, we ensure that analyses can still be conducted even with incomplete data.

This raises the question of whether a fully or partially time-explicit representation of supply chains may be sufficient, as additional model complexity does not always result in more insights, see discussion above for the case study. Pinsonnault et al. (2014) found that considering the temporal distribution of the entire supply chain with dynamic LCIA led to over 10% deviation in GWI for 8.6% of the products in ecoinvent v2.2. Including temporal evolution in inventories would likely result in even greater deviations. However, a time-explicit approach to entire supply chains might be infeasible due to time and data constrains. Collet et al. (2014) suggest to focus on processes above a certain threshold and those, for which the timescale of processes and emissions matches or exceeds that of the impact category. When it is shorter, averages can be used, as values will naturally aggregate to the coarser timescale of the impact category. Shimako et al. (2018) have found that the sensitivity to temporal resolution for both LCI and LCIA is impact-category-dependent, finding a high sensitivity to step size for both LCI and LCIA for toxicity impacts, and a low sensitivity for climate change impacts. These findings could inform the choice of an appropriate temporal resolution in time-explicit LCA, taking into account not only the temporal sensitivities of the impact category and product system, but, relevant in our approach, also the timescale of the time-specific background databases as an additional criterion.

Although time-explicit LCA marks a significant step towards a more nuanced representation of temporal dynamics in LCA, it still only accounts for the time-specific fractions of processes and emissions tied to the fulfilment of the functional unit, excluding emissions from other economic activities at the same timestep. This highlights that, like dLCA or any form of LCA, time-explicit LCA is not suitable as a substitute for risk assessment (Guinée et al. 2017) or for application in absolute environmental assessments (Guinée et al. 2022).

The implementation in the *bw_timex* package has some additional technical constraints. Currently, temporal convolution halts at the background database, restricting temporalization to the foreground system, while previous research has shown that background temporalization can be important (Pinsonnault et al. 2014). This restriction can be bypassed by moving (parts of) the background supply chain, ideally those containing the highest-contributing flows, into the foreground system. Moreover, emissions of background supply chains are aggregated at the temporal markets. This modeling choice was made to preserve the timing of the emissions. Cardellini et al. (2018) show that best-first graph traversal is effective for dLCA, but applying it to time-explicit LCA introduces a challenge: Changes within supply chains can shift the prioritization of branches, and since the exact time-explicit supply chains are unknown during the initial graph traversal, important branches may be excluded based on the defined cutoff threshold. To avoid this, a sufficiently low cutoff threshold should be selected.

In the presented approach, we preserve the traditional matrix-based LCA structure to ensure compatibility with existing frameworks and tools. Alternative methods for integrating time are feasible, particularly when moving beyond the conventional matrix structure. Instead of adding the temporal information as new row/column pairs to the $A$ and $B$ matrices, time could be incorporated as an additional dimension in the vectors and matrices. This could be done from the outset, turning the $A$ and $B$ matrices into three-dimensional tensors and preserving this time dimension throughout inventory calculations. Alternatively, a time dimension can be introduced after determining the supply vector conventionally. The temporal information of when a process is supplied can be retrieved from the timeline of intermediate processes, yielding a supply matrix. Element-wise multiplication of each time-slice of the supply matrix with the biosphere matrix of that point in time yields a three-dimensional time-explicit inventory tensor.

Lastly, the computational time of a time-explicit LCA is generally higher than that of a standard LCA, though the extent depends on the system studied. In general, the number of necessary computations in a time-explicit LCA scales with the number of temporalized intermediate flows that must be traversed. This number grows linearly with the number of processes per supply chain tier, but exponentially with the number of tiers and the number of time steps in the rTDs. Each time step creates a new “virtual temporal branch” in the supply chain that needs to be followed to reach downstream processes. Benchmarking with *bw_timex* v0.3.1 (Diepers et al. 2025b) shows that for a test system with 4 Supply chain tiers, 5 processes per tier (fully connected across tiers), and 2 time steps per rTD, the total calculation time is 82 ± 2 s (mean and standard deviation of 3 runs using an Apple M4 Pro processor). As a comparison, for the same system with 10 time steps per rTD, the total calculation time is 865 ± 8 s. Additional benchmarking results are available on GitHub.

### Link to spatial LCA

As Heijungs and Suh (2002) point out, the implementation of temporal differentiation has a strong resemblance to spatial differentiation in LCA, which is a common practice for both LCI and LCIA data (Frischknecht et al. 2019; Mutel et al. 2019; Shi and Yan 2024). Existing LCI databases commonly feature separate regional processes (e.g., electricity production in Spain and Italy) (Wernet et al. 2016), which is similar to the separate temporal processes in our approach (e.g., electricity production in 2023 and 2024). Regional markets group spatially-specific processes, much like our temporal markets bridge different time periods. Similarly, elementary flows are spatially distinguished, i.e. emissions to “urban air close to ground” and “non-urban air or from high stacks” (Wernet et al. 2016), and can be paired with spatially specific characterization factors (Mutel et al. 2019).

Spatial differentiation has received considerable attention in the LCA community, with various computational solutions proposed (Maier et al. 2017; Li et al. 2021; Mutel and Hellweg 2023; Peng and Pfister 2024). For instance, Peng and Pfister (2024) introduced a database-wide regionalization of activity datasets using trade data from a multi-regional input-output model. While such “fully regionalized” databases could be directly applied in a time- and region-explicit LCA using *bw_timex*, the level of temporal and regional resolution needs to be carefully selected to fit the research question while balancing the additional computational complexity. As for temporalization (Collet et al. 2014), prioritization is also necessary for regionalization (Patouillard et al. 2019). Future research could therefore explore adaptive frameworks that leverage contribution and uncertainty analysis to determine where a selective application of regional and temporal detail is most impactful, while maintaining higher levels of spatial and temporal aggregation for less critical parts of the supply chain.

### Link to MFA

LCA is often used in combination with material flow analysis (MFA), whether static or dynamic (Pauliuk and Hertwich 2016; Barkhausen et al. 2023). While a comprehensive discussion of system dynamics approaches is beyond the scope of this paper, we briefly highlight how time-explicit LCA can interface with dynamic MFA (dMFA). Time-explicit LCA, with its temporally distributed supply chains and the consideration of the specific technology landscape at each point in time, offers significant opportunities for integration with dMFA. An integrated dMFA and time-explicit LCA allows the analysis of material flows within and into the foreground system, while also calculating the elementary flows and impacts. Unlike classical dMFA software such as *ODYM* (Pauliuk and Heeren 2019), which is typically stock- or inflow-driven, *bw_timex* currently only supports outflow (or final demand) driven models. Additionally, while *bw_timex* automatically produces material flows and impacts, the stock levels and changes must be derived separately from the timeline of intermediate flows. Typical features of dMFA are the use of lifetime distributions and age cohorts. In *bw_timex*, lifetime distributions can be modelled using rTDs, while differing age cohorts are represented by distinct products and producing activities at different timesteps, e.g., years. This makes *bw_timex*, particularly when combined with a modular LCA approach as proposed in Steubing et al. (2016), a powerful tool for combined dMFA-LCA assessments.

## Conclusion

Time-explicit LCA represents a significant advancement in accounting for temporal dynamics in LCA by jointly considering temporal distribution and temporal evolution of processes, emissions, and environmental responses at the LCI and LCIA stage. The resulting time-explicit inventory records the emissions as they occur in time, reflecting the technology landscape at each point in time. This time-explicit framework enables more representative modeling of the emissions across the life cycle of the product under study. It is especially valuable for assessing long-lived products and supply chains with temporal variability, particularly when applied in scenarios that envision transformative technological changes.

An implementation of the time-explicit LCA framework is available as the open-source python package *bw_timex *(Diepers et al. 2025b) within the *Brightway* LCA ecosystem (Mutel 2017), with seamless integration for prospective databases generated via *premise* (Sacchi et al. 2022). The tool automatically propagates rTDs through the supply chain and links intermediate flows to time-specific databases according to their time of occurrence, offering substantial time savings and scalability improvements compared to manual approaches. The implementation supports a high degree of customization of the data inputs, accommodating the different temporal requirements across impact categories and the varying availability of time-specific data. Built on the conventional LCA matrix structure, *bw_timex* is also compatible with other LCA tools. We apply the framework in a case study of an EV, showcasing significant differences between the time-explicit results and the results of assessments that model all processes at a single point in time. As temporal information is preserved in the LCI, dynamic LCIA methods can be applied, which we demonstrate for climate change impacts.

Further research may include coupling time-explicit LCA with dynamic MFA or spatial LCA, and filling data gaps to enable time-explicit LCAs for entire supply chains and for impact categories besides climate change.

## Data Availability

The source code of the software *bw_timex* can be accessed via the GitHub repository at: https://github.com/brightway-lca/bw_timex. Extensive documentation of *bw_timex* is available at: https://docs.brightway.dev/projects/bw-timex/en/latest/. The EV case study notebook can be accessed at: https://docs.brightway.dev/projects/bw-timex/en/latest/content/examples/paper_case_study.html. The notebook for benchmarking calculation time can be accessed at: https://github.com/brightway-lca/bw_timex/blob/main/notebooks/run_time_test_benchmarking.ipynb
